# Reduced Affiliative Behavior in Autism Reflects Greater Dependence on Perceived Similarity

**DOI:** 10.21203/rs.3.rs-9281318/v1

**Published:** 2026-04-10

**Authors:** Yu Hao, Alessandra N.C. Yu, Sarah M. Banker, Matthew Schafer, Ember Zhang, Sarah Barkley, Jadyn Trayvick, Arabella W. Peters, Abigaël A. Thinakaran, Christopher McLaughlin, Xiaosi Gu, Jennifer H. Foss-Feig, Daniela Schiller

**Affiliations:** 1. Department of Psychology, Ningbo University, Ningbo, Zhejiang, China; 2. Nash Family Department of Neuroscience, Icahn School of Medicine at Mount Sinai, New York, NY, USA; 3. Department of Psychiatry, Icahn School of Medicine at Mount Sinai, New York, NY, USA; 4. Seaver Autism Center for Research and Treatment, Icahn School of Medicine at Mount Sinai, New York, NY, USA; 5. Center for Computational Psychiatry, Icahn School of Medicine at Mount Sinai, New York, NY, USA; 6. Friedman Brain Institute, Icahn School of Medicine at Mount Sinai, New York, NY, USA; 7. Department of Child and Adolescent Psychiatry, NYU Grossman School of Medicine; 8. School of Medicine, Columbia University; 9. School of Engineering and Applied Science, University of Pennsylvania, Philadelphia, PA, USA; 10. Department of Psychology, Stony Brook University; 11. Department of Psychology, Montclair State University; 12. Columbia University Irving Medical Center; 13. Psychiatry and Biomedical Informatics & Data Science, Yale University; 14. Mindich Child Health and Development Institute, Icahn School of Medicine at Mount Sinai

**Keywords:** autism spectrum disorder, similarity attraction, empathy, hippocampus, posterior cingulate cortex

## Abstract

Social difficulties in autism are often framed as reduced motivation, yet this account does not explain when and why autistic individuals affiliate. We show that autism selectively alters the architecture—not the presence—of similarity-based social behavior. Across two independent samples (online, n = 714; in-person, n = 225), autistic and neurotypical adults exhibited comparable context-dependent (selective) preference for relatively similar others. In contrast, autistic individuals showed a markedly stronger global coupling between perceived similarity and affiliative behavior, such that low perceived similarity was associated with sharply reduced affiliation. This effect was strongest among those with lower trait empathy. Structural MRI revealed dissociable contributions of hippocampal and posterior cingulate cortex volumes to this coupling across groups. These findings demonstrate that autism preserves similarity attraction while amplifying its role as a stable heuristic for social engagement, supporting a model in which social motivation is restructured toward similarity-dependent engagement rather than diminished.

The need to affiliate—to associate or interact with others—is fundamental to human survival, mental health, and well-being^1,2^. However, individuals vary not only in how affiliation is expressed across contexts and social targets, but also in their overall propensity to affiliate^3,4^. Individuals with autism spectrum disorder (ASD) are often described as exhibiting diminished social motivation^5^, reduced affiliative behavior^6^ and atypical self-other processing^7–9^. Emerging work suggests that social engagement in autism may depend more strongly on perceived similarity or shared identity. For example, individuals with ASD have been shown to empathized more readily with others who resemble themselves^10^, a phenomenon that extends to helping motivation when engaging with other people with ASD^11^. This apparent paradox has led to growing recognition that social connection in autism may be governed by alternative mechanisms rather than deficient ones^12,13^.

People tend to move toward and remain close to others who are similar to themselves^14,15^. This similarity attraction tendency is a robust principle in social psychology^16,17^. Interacting with similar others helps individuals build more accurate models of others and their relationship^18^, reducing anxiety and facilitating communication, cooperation, and trust^14,15,19,20^. However, affiliation based solely on similarity can also be limiting: it may increase segregation between groups, reduce social mobility, and constrain epistemic diversity^14,20^. Although structural factors such as geography or institutions can amplify these patterns, preferences for similarity often arise from shared attitudes, beliefs, or perceived identity^15,21^. Against this backdrop, autism presents a key puzzle: not whether affiliation is reduced, but how it is organized. We propose that affiliation in ASD may be more strongly gated by perceived similarity, and leverage similarity–affiliation coupling processes to test this across levels of analysis.

Similarity–affiliation coupling may operate at multiple levels. At a context-specific selective level, individuals preferentially affiliate with targets perceived as relatively more similar than others in a given context—a phenomenon well established in the similarity attraction literature^15^. Prior work has further shown that the strength of this coupling depends on how individuals construe relationships, interpret shared attitudes, represent the self in relation to others and perceived control ^18,22–25^. At a global level, individuals who generally perceive others as more similar may also exhibit higher overall affiliative tendencies. These levels are theoretically distinct: the former reflects dynamic, context-sensitive selection processes, whereas the latter reflects a more stable social-perceptual orientation. Prior work suggests that autism is associated with reduced context sensitivity in decision-making, including diminished integration of contextual and emotional cues^26,27^. At the same time, autistic individuals often show a preference for predictability and may rely more on stable heuristics to guide social behavior^28^. Together, these patterns raise the possibility that similarity-affiliation processes may be expressed differently across the selective and global levels in ASD. However, these levels have rarely been distinguished or examined simultaneously within a single paradigm, limiting our understanding of how affiliative behavior is structured in autism.

In our study, we examined two psychological factors that may differentially shape selective and global similarity–affiliation coupling in autism: perceived control and empathic resonance. Perceived control may preferentially influence context-dependent selective similarity-attraction^22^. Similar others are typically easier to predict and understand, thereby reducing uncertainty and cognitive load^15^. When individuals experience lower perceived control in social interactions, affiliating with similar others may restore a sense of structure and predictability ^29,30^. Thus, perceived control may moderate the extent to which perceived similarity guides moment-to-moment affiliative decisions. Second, empathic resonance may be more closely tied to global similarity–affiliation coupling. From an evolutionary perspective, reflexive sensitivity to others’ emotions and intentions may represent an ancestral form of empathy that supports affiliative bonding and social fitness^31^. Thus, individual differences in empathy may not merely reflect variation in cognitive mentalizing ability but may index the motivational and affective substrate through which similarity cues are translated into affiliative behavior. Research has shown that empathy may facilitate a richer mental simulation of another’s inner world^32,33^, transforming similarity into felt closeness, and making the connection feel more intuitive and rewarding^34^. Given the well-documented variability in empathic traits in ASD^35^, empathy may differentially calibrate global similarity–affiliation alignment within the autistic population.

Neurally, a key region that may support similarity-affiliation coupling is the hippocampus, which has long been recognized for its role in relational memory—linking experiences, people, and contexts (e.g.,^36,37^). Beyond its function related to memory, converging evidence suggests that the hippocampus contributes to social behavior by forming and reconstructing relational representations (who did what, when, and in what context) that can be flexibly recombined to guide decisions, perspective-taking, and affiliation (e.g.,^38–41^). In addition, the posterior cingulate cortex (PCC)—a core node of the default mode and mentalizing networks—has been implicated in representing self-relevant information and tracking social distance during ongoing interactions in both ASD and non-ASD samples^41–45^. If the PCC provides a distance metric that helps regulate approach–avoid decisions, then individual differences in PCC may calibrate how strongly perceived similarity (a cue that compresses social distance) influences affiliative behavior. Given relevance of hippocampus and PCC in ASD and its social related phenotypes^46–49^, we conceptualized the volumes of these regions as relatively stable trait-level neural features that may differentially relate to global similarity–affiliation alignment, reflecting enduring differences in relational representation and self–other processing.

Integrating behavioral, psychological, and neural evidence, our study tested whether similarity–affiliation coupling operates differently at the selective and global levels in ASD compared to neurotypical (NT) individuals. Rather than assuming uniform alteration, we examined whether autism would be characterized by changes in selective similarity-attraction, global similarity–affiliation alignment, or both. Using an ongoing, naturalistic social navigation task^41^(Fig. 1A) to derive both levels of similarity-affiliation coupling (Fig. 2B), we first tested group differences (Fig. 1C) and then examined whether perceived control, trait empathy, and hippocampal and PCC volumes would moderate these processes across groups (Fig. 1D–F). By moving beyond descriptive effects to uncover dissociable mechanisms, this work aims to provide a novel framework for understanding the architecture of autistic social affiliative behaviors.

## Results

### Characteristics of samples and demonstration of similarity-affiliation coupling in ASD with our social navigation task

Our study incorporated three independent samples that varied in demographic composition (e.g., sex ratio, education level), self-perceived autistic traits, data collection method (online vs. in-person), and developmental stage. Although not all measures were available in every sample, this multi-sample design enabled cross-validation of key findings across complementary datasets.

In the online adult sample (for demographics, see Table 1A), participants were young adults with self-report of having a professional diagnosed ASD (n = 357, sample removed those who reported cognitive delays in order to match NT controls) and NT participants matched on age, sex and education (n = 357). Compared with the online adult sample, the in-person adult sample (ASD n = 103, NT n = 122; for demographics, see Table 1B) was significantly older, had higher educational attainment, a greater proportion of males, lower self-rated autism symptoms, and lower self-rated social avoidance in both the ASD and NT groups (all ps < 0.01). The in-person participants were screened for ASD by licensed clinicians. The online adolescent ASD sample (demographics see Table 1C) consisted of adolescent participants with parent-reported professional ASD diagnoses (n = 445, including those with cognitive delays).

**Figure 1.**
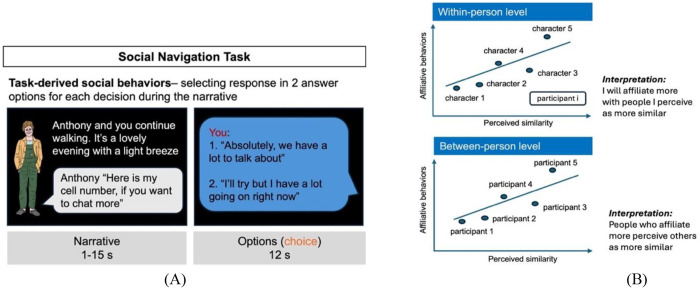

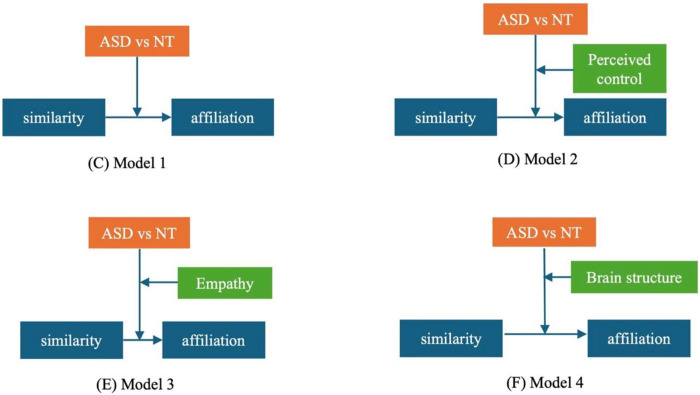
Experimental paradigm and conceptual models of research questions. **(A)** Participants engaged in a dynamic, role-playing game simulating social scenarios. Illustrated characters with distinct social roles (e.g., an old acquaintance or potential employer) appeared on slides. The participant engages in repeated interactions with the same characters, allowing the relationships to evolve. Unlike traditional paradigms that rely on discrete, one-off ratings (e.g., choosing a partner or expressing preference for similar others), our task captures implicit, dynamic social behaviors that more closely approximate real-world interaction. The affiliation trials in the task represent different scenarios of physical contact/social engagement throughout a storyline. There are an affiliation choice (scored +1) and a non-affiliation one (scored −1), with scores computed unbeknownst to the participants. For example, in the affiliation trial shown in the figure, response 1 to “Anthony” is recorded as +1, while response 2 is recorded as −1 (for other example trials see Methods). Across the task, participants interacted with 5 different social characters, completing 6 trials per character, resulting in 30 affiliation trials, which were intermixed with 30 power trials representing either authoritative or submissive roles (see Methods for details). After the task, participants rated their perceived affiliation with each character based on friendship and intimacy. Participants also rated their perceived similarity to each character, as well as their perceived social control during social interaction by rating how much impact they felt they had on each character during the interaction. **(B)** Conceptual illustration of multi-level similarity-affiliation coupling. Affiliative behavior was first averaged across the six trials within each character to obtain a within-person measure. These character-level values were then averaged across all five characters to derive a between-person measure of affiliative tendency. Selective similarity-attraction (within-person effect): affiliation increases for characters perceived as relatively more similar within an individual. Global similarity-affiliation alignment (between-person effect): individuals who affiliate more overall also perceive others as more similar overall. **(C)** Model 1 tests whether autism status (ASD vs. NT) moderates similarity-affiliation coupling. **(D)** Model 2 tests whether the moderating role of perceived control on similarity-affiliation coupling differs between ASD and NT groups. **(E)** Model 3 tests whether the moderating role of trait empathy on similarity-affiliation coupling differs between ASD and NT groups. **(F)** Model 4, similar to Model 2 and 3, examines brain regions of interest (ROI: hippocampus and posterior cingulate cortex) as moderators.

Prior to testing Model C-F (Fig. 1), we confirmed that individuals with ASD exhibited significantly lower affiliative behaviors, as measured by our social navigation task, than NT individuals in both online and in-person adult samples (ps < 0.001; Table 1), consistent with established ASD phenotypes characterized by atypical patterns of affiliation^5^. Moreover, we found similarity-affiliation coupling at both selective and global levels in our three ASD samples (ps < 0.001), replicating and extending prior findings from non-ASD populations ^18,22–25^.

### Similarity-affiliation coupling is stronger in ASD compared to NT at the global (between-person) but not selective (within-person) level

**Figure 2.**
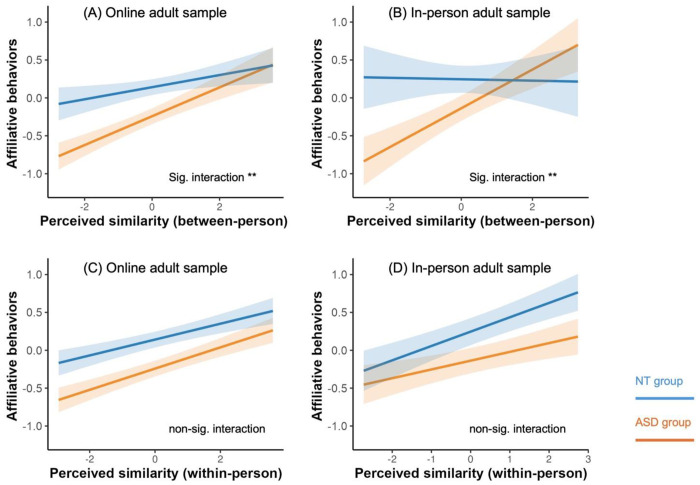
Similarity-affiliation coupling was significantly stronger in the ASD group than in the NT group at the global (between-person) level (A, B), whereas selective (within-person) similarity-attraction did not differ between groups (C, D). Within-person and between-person effects were estimated simultaneously within the same mixed-effects model for each sample. In the figures, predicted values were derived from the models with covariates held constant (continuous covariates at their mean and categorical covariates at their reference levels). Shaded regions represent pointwise 95% confidence intervals around the estimated lines. Notation: *** p < 0.001, ** p < 0.01, * p < 0.05. **(A)** Between-person effect differed between ASD (n = 357) and NT (n = 357) groups in the online adult sample. Simple slopes analyses showed a stronger similarity-affiliation coupling in the ASD group (β = 0.19, 95% CI [0.13, 0.25]) than in the NT group (β = 0.08, 95% CI [0.02, 0.14]). **(B)** Between-person effect differed between ASD (n = 103) and NT (n = 122) groups in the in-person adult sample. Simple slopes analyses showed a stronger similarity-affiliation coupling in the ASD group (β = 0.26, 95% CI [0.16, 0.35]) than in the NT group ( β = −0.01, 95% CI [−0.14, 0.13]). **(C)** Within-person effect did not differ between ASD (β = 0.14, 95% CI [0.10, 0.18]) and NT (β = 0.11, 95% CI [0.06, 0.15]) groups in the online adult sample. **(D)** Within-person effect did not differ between ASD (β = 0.12, 95% CI [0.05, 0.18]) and NT (β = 0.19, 95% CI [0.12, 0.26]) groups in the in-person adult sample.

**Figure 3.**
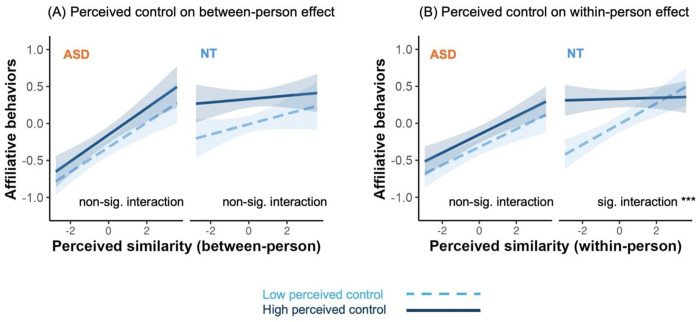
Lower perceived control was associated with stronger selective (within-person) similarity-attraction in the NT group, but not in the ASD group, whereas no moderation effect was observed at the global (between-person) level. Figures show data from the online adult sample (ASD n = 357, NT n = 357). Within-person and between-person effects were estimated simultaneously within the same mixed-effects model. In the figures, predicted values were derived from the model with covariates held constant (continuous covariates at their mean and categorical covariates at their reference levels). Model-estimated associations were shown at higher (+1 SD; solid lines) and lower (−1 SD; dashed lines) levels of the perceived control. Shaded regions indicate pointwise 95% confidence intervals around the estimated lines. Notation: *** p < 0.001, ** p < 0.01, * p < 0.05. **(A)** Simple slopes analyses showed that the between-person effect was comparable across levels of perceived control in the ASD group (βs ≥ 0.16, 95% CIs [0.08, 0.24]). In the NT group, although stronger between-person effect emerged at lower perceived control (β = 0.08, 95% CIs [0.00, 0.16]) not at higher perceived control (β = 0.03, 95% CIs [0.04, 0.10]), the interaction term was not significant. **(B)** Simple slopes analyses showed that the within-person effect was again comparable across levels of perceived control in the ASD group (βs ≥ 0.09, 95% CIs [0.04, 0.16]). In contrast, in the NT group, stronger within-person effect emerged at lower perceived control (β = 0.17, 95% CI [0.11, 0.23]), but not at higher perceived control (β = 0.04, 95% CI [−0.01, 0.09]), showing a signiciant interaction.

Model 1 was first tested in the online adult sample and subsequently replicated in the in-person adult sample. A multilevel mixed-effects model was used to simultaneously estimate selective (within-person) similarity attraction and global (between-person) similarity-affiliation alignment, including interaction terms for within-person similarity ^ group and between-person similarity × group in predicting affiliative behavior (statistical model details see Methods). We found that at the between-person level, the similarity-affiliation alignment was significantly stronger in the ASD group than in the NT group in the online adult sample (β = −0.11, S.E. = 0.04, ΔR^2^ (semi-partial R^2^) = 0.005, χ^2^ = 6.74, p = 0.010, Fig. 2A). This pattern was replicated in the in-person adult sample (β = −0.26, S.E. = 0.08, ΔR^2^ = 0.020, χ^2^ = 9.89, p = 0.002, Fig. 3B). In contrast, the within-person level did not differ between groups in both online (β = −0.04, S.E. = 0.03, χ^2^ = 1.61, p = 0.205) and in-person adult sample (β = 0.07, S.E. = 0.05, χ^2^ = 2.55, p = 0.110). The point estimates in both samples even reversed direction, further suggesting the absence of a reliable group difference.

These findings indicate that despite lower overall affiliation in ASD, selective similarity-attraction operated comparably in ASD and NT individuals. In contrast, at the global level, the affiliative behavior in ASD was more tightly gated by perceived similarity tendency, such that individuals with ASD who perceived others as dissimilar showed disproportionately low affiliation, whereas those who perceived others as similar showed affiliation levels comparable to NT individuals.

Exploratory analysis extending to self-perceived autistic traits indicated that the stronger global similarity-affiliation alignment was not specific to clinically diagnosed ASD but also shown in those with higher self-rated autistic traits (online adult sample p = 0.001 and in-person adult sample p = 0.047). In terms of the selective similarity-attraction, autistic traits didn’t reach consistent findings across samples (online adult sample p = 0.030; and in-person adult sample p = 0.221). Given their exploratory nature and inconsistent patterns, these results are not interpreted further.

### Similarity-affiliation coupling is moderated by perceived control in NT but not ASD at the selective (within-person) level

Model 2 was tested in the online adult sample and was replicated in the online adolescent sample. Perceived control in our study was operationalized as perceived impact on others in the task, reflecting whether one’s actions meaningfully influence the interaction. We found that the global (between-person) similarity-affiliation alignment was not moderated by perceived control in both groups (ps > 0.247) and did not differ by groups (p = 0.302) (Fig. 3A). However, the moderating effect of perceived control on the selective (within-person) similarity attraction differed between groups in the online adult sample, as indicated by a significant perceived control × within-person similarity × group three-way interaction (β = −0.07, S.E. = 0.03, χ^2^ = 5.92, *p* = 0.015; Fig. 3B). Specifically, among NT individuals, lower perceived control predicted stronger selective similarity-attraction effect (β = −0.07, S.E. = 0.02, ΔR^2^ = 0.009, χ^2^ = 12.93, *p* < 0.001), which was consistent with Ma et al. (2025). In contrast, this effect was absent in ASD participants (β = 0.00, S.E. = 0.02, ΔR^2^ = 0.000, χ^2^ = 0.01, *p* = 0.931).

To quantify evidence for practical absence of the effect in ASD, using a Bayesian multilevel model, we again observed a group-specific moderation of selective similarity attraction by perceived control. In the NT group, there was a reliable interaction between within-person perceived similarity and perceived control in predicting affiliative behavior (posterior median β = −0.07, 95% credible interval [−0.106, −0.026]). Although modest in magnitude, this interaction was unlikely to be practically negligible: only 22.7% of the posterior mass fell within a predefined region of practical equivalence (ROPE; ±0.05 on standardized coefficients). In contrast, the corresponding interaction in the ASD group was effectively absent. The posterior median for the interaction was close to zero (β = 0.00), with a 95% credible interval spanning zero [−0.037, 0.039]. Critically, 98.9% of the posterior mass lay within the ROPE, and an interval-null Bayes factor strongly favored practical equivalence over a non-trivial effect (BF01 = 608), providing decisive evidence that perceived control did not meaningfully moderate selective similarity attraction in ASD.

Consistent with these patterns, the three-way interaction between perceived control, within-person perceived similarity, and group was credibly non-zero. The posterior median for the group difference (ASD - NT) was 0.07, with a 95% credible interval of [0.013, 0.121]. Only 28.3% of the posterior mass fell within the ROPE, and BF_01_ was 2.59, which indicated that the data leaned toward a small effect but do not conclusively rule out a non-trivial difference.

Furthermore, we replicated the null effect of the perceived control on the selective similarity attraction to the online adolescent ASD sample (β = −0.03, S.E. = 0.02, ΔR^2^ = 0.00, χ^2^ = 2.27, *p* = 0.132). The within-person perceived control × similarity interaction was again practically negligible (median β = −0.03, 95% credible interval [−0.059, 0.007]). We found that 93% of the posterior distribution fell within the ROPE, and interval-null Bayes factor analysis provided strong evidence for practical equivalence (BF_01_= 82). Together, these results show that perceived control selectively shaped within-person similarity attraction in NT individuals, reflecting a context-dependent effect, whereas this effect was absent in ASD across adolescents and adults.

### Similarity-affiliation coupling is moderated by trait empathy in ASD but not NT at the global (between-person) level

**Figure 4.**
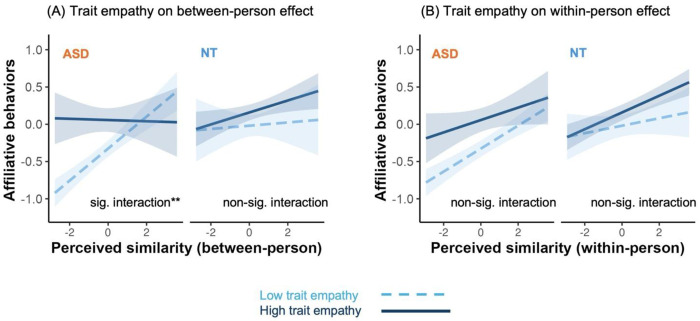
Lower trait empathy was associated with stronger global (between-person) similarity-affiliation alignment in the ASD group, but not in the NT group, whereas no moderation was observed at the selective (within-person) level. Figures show data from the online adult sample (ASD n = 357, NT n = 357). Within-person and between-person effects were estimated simultaneously within the same mixed-effects model. In the figures, predicted values were derived from the model with covariates held constant (continuous covariates at their mean and categorical covariates at their reference levels). Model-estimated associations were shown at higher (+1 SD; solid lines) and lower (−1 SD; dashed lines) levels of the trait empathy. Shaded regions indicate pointwise 95% confidence intervals around the estimated lines. Notation: *** p < 0.001, ** p < 0.01, * p < 0.05. **(A)** Simple slopes analyses showed that the between-person effect emerged at lower trait empathy in the ASD group (β = 0.21, 95% CI [0.14, 0.27]), but not at higher trait empathy (β = −0.01, 95% CI [−0.14, 0.12]), showing a significant intearction. In contrast, in the NT group, although the between-person effect emerged at higher trait empathy (β = 0.08, 95% CI [0.02, 0.14]) not at lower trait empathy (β = 0.02, 95% CI [−0.10, 0.15]), the interaction term was not significant. **(B)** Simple slopes analyses showed that the within-person effect emerged at lower trait empathy in the ASD group (β = 0.13, 95% CI [0.09, 0.18]) but not at higher trait empathy (β = 0.05, 95% CI [−0.05, 0.15]). In contrast, in the NT group, the within-person effect emerged at higher trait empathy (β = 0.14, 95% CI [0.10, 0.18]) but not at lower trait empathy (β = 0.08, 95% CI [−0.01, 0.16]). However, the interaction term was not significant in either group.

Model 3 was tested across all 3 samples. We administered the Empathy Quotient questionnaire as a measure of trait empathy that has been validated in ASD samples ^50^. We found that the global (between-person) similarity-affiliation alignment was moderated by trait empathy differentially by groups in the online adult sample, indicated by a significant empathy × between-person similarity × group three-way interaction (β = 0.14, S.E. = 0.05, χ^2^ = 7.82, p = 0.005; Fig. 4A). Specifically, in the NT group, trait empathy did not moderate the between-person effect (β = 0.03, S.E. = 0.03, ΔR^2^ = 0.000, χ^2^ = 0.69, p = 0.407), while among ASD individuals, those with lower trait empathy showed stronger global similarity-affiliation alignment than those with higher empathy (β = −0.11, S.E. = 0.04, ΔR^2^ = 0.011, χ^2^ = 9.05, p = 0.003). However, we did not find significant selective (within-person) similarity attraction effect in both groups (ps > 0.129) and it did not differ by groups (p = 0.066) (Fig. 4B).

The different moderation effects of empathy for ASD vs NT groups were replicated in the in-person adult sample, showing a significant empathy × between-person similarity × group three-way interaction (β = 0.27, S.E. = 0.10, χ^2^ = 7.85, p = 0.005). Specifically, in the NT group, trait empathy did not moderate the between-person effect (β = 0.03, S.E. = 0.03, ΔR^2^ = 0.013, χ^2^ = 0.74, p = 0.391); while among ASD individuals, those with lower trait empathy showed stronger global (between-person) similarity-affiliation alignment than those with higher empathy (β = −0.11, S.E. = 0.04, ΔR^2^ = 0.020, χ^2^ = 8.62, p = 0.003). Again, we did not find significant selective (within-person) similarity attraction effect in both groups (ps > 0.238) and it did not differ by groups (p = 0.108).

We conducted a series of robustness analyses, including sensitivity and specificity tests. First, the empathy moderation effect observed in the ASD group extended to ASD individuals with cognitive delays. In the online adult sample, when ASD participants with cognitive delays were included (total n = 575), empathy continued to moderate the between-person effect (β = −0.13, S.E. = 0.03, p < 0.0001), whereas the presence of cognitive delays themselves did not influence this effect (Cognitive delay × group two-way interaction, p = 0.932). These findings suggest that empathy effect holds regardless of cognitive functioning differences. Second, to examine specificity of this effect, we examined whether trait empathy moderated similarity-affiliation coupling as a function of self-rated autistic severity across participants and observed null effect in both online and in-person adult sample (ps > 0.131). Together, these findings suggest that the empathy-related moderation of similarity-affiliation coupling may be specific to individuals with an ASD diagnosis rather than reflecting elevated autistic traits.

In addition, we also examined whether trait empathy moderated similarity-affiliation coupling in the online adolescent ASD sample. Trait empathy did not moderate global (between-person) similarity-affiliation alignment (β = 0.01, S.E. = 0.03, χ^2^ = 0.05, p = 0.818). Instead, it significantly moderated selective (within-person) similarity-attraction (β = 0.03, S.E. = 0.02, χ^2^ = 4.03, p = 0.045). Specifically, adolescents with ASD who had lower trait empathy showed a stronger tendency to affiliate with targets they perceived as more similar to themselves. Because we did not include NT adolescent controls, we were unable to determine whether this moderation pattern is specific to ASD or reflects a broader developmental phenomenon.

### Similarity-affiliation coupling is differentially moderated by hippocampus and PCC volume in ASD and NT at the global (between-person) level

**Figure 5.**
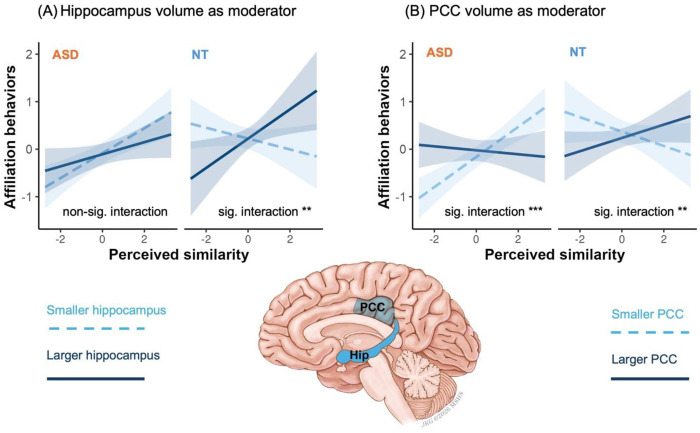
Hippocampal and PCC volumes were differentially associated with global (between-person) similarity-affiliation alignment across groups. Larger hippocampal volume was associated with stronger global alignment in the NT group but not in the ASD group (A). PCC volume showed a comparable pattern as hippocampus in the NT group, whereas in the ASD group, smaller PCC volume was associated with stronger global alignment (B). Figures show data from the in-person adult sample with available neuroimaging data (ASD n = 72, NT n = 102). Separate mixed-effects models were conducted for hippocampus and PCC as moderators of global similarity-affiliation alignment. Predicted values were derived from the models with covariates held constant (continuous covariates at their mean and categorical covariates at their reference levels). Model-estimated associations were shown at higher (+1 SD; solid lines) and lower (−1 SD; dashed lines) levels of reginal brain volume. Shaded regions indicate pointwise 95% confidence intervals around the estimated lines. Notation: *** p < 0.001, ** p < 0.01, * p < 0.05. **(A)** Hippocampal volume. In the NT group, the between-person effect emerged at larger hippocampal volume (β = 0.33, 95% CI [0.10, 0.55]) but not at smaller volume (β = −0.12, 95% CI [−0.28, 0.05]). In the ASD group, although the between-person effect emerged at smaller hippocampal volume (β = 0.27, 95% CI [0.10, 0.43]) not at larger volume (β = 0.09, 95% CI [−0.09, 0.26]), the interaction term was not significant. **(B)** PCC volume. In the NT group, the between-person effect emerged at larger PCC volume (β = 0.16, 95% CI [0.01, 0.31]) but not at smaller volume (β = −0.19, 95% CI [−0.38, 0.01]). In the ASD group, the between-person effect emerged at smaller PCC volume (β = 0.30, 95% CI [0.17, 0.44]) but not at larger PCC volume (β = −0.08, 95% CI [−0.26, 0.11]). The interaction term was significant in both groups.

Finally, Model 4 was tested in the in-person adult sample. We found that hippocampal volume differentially moderated the global (between-person) similarity-affiliation alignment across groups in the online adult sample, indicated by a significant hippocampus × between-person similarity × group three-way interaction (β = 0.28, S.E. = 0.10, χ^2^ = 8.26, p = 0.004, Fig. 5A). In the NT group, larger hippocampal volume predicted stronger between-person effect (β = 0.22, S.E. = 0.07, ΔR^2^ = 0.026, χ^2^ = 9.19, p = 0.002), whereas in the ASD group, hippocampal volume did not show a significant moderation effect (trending the opposite direction, β = −0.09, S.E. = 0.06, ΔR^2^ = 0.014, χ^2^ = 2.58, p = 0.108). In contrast, selective (within-person) similarity attraction effect was not significant in both groups (ps > 0.096) and did not differ by groups (p = 0.054). Controlling for intracranial volume did not change the above conclusions.

A similar pattern was observed for PCC volume, which also showed a significant PCC × between-person similarity × group three-way interaction (β^2^= 0.32, S.E. = 0.08, χ^2^ = 16.80, p < 0.001, Fig. 5B). Paralleling the hippocampal findings, in the NT group, larger PCC volume predicting stronger between-person effect (β = 0.17, S.E. = 0.06, ΔR^2^ = 0.022, χ^2^ = 8.38, p = 0.004). In the ASD group, the opposite trend was observed—smaller PCC volume predicted stronger between-person effect (β = -0.19, S.E. = 0.05, ΔR^2^ = 0.065, χ^2^ = 14.87, p < 0.001). Although our analysis was an ROI-based test, PCC findings were the only significant region for whole brain analysis corrected by family-wise comparisons. Again, selective (within-person) similarity attraction effect was not significant in both groups (ps > 0.270) and did not differ by groups (p = 0.254). Controlling for intracranial volume did not change the above conclusions.

We explored whether these findings were specific to ASD or also applied to high self-perceived autistic traits. We found that hippocampal moderation effect did not differ across self-rated autism symptoms levels on both within- and between-person effect (ps > 0.242). These results suggest that the ASD vs NT group differences for the hippocampus volume moderation on global similarity-affiliation alignment was unique to clinically ascertained ASD diagnosis and unrelated to self-perceived autistic traits. On the other hand, for PCC volume, the pattern of self-rated autism symptoms resembled that of ASD: individuals with higher self-reported autistic traits showed stronger similarity attraction when PCC volume was smaller (β = −0.09, S.E. = 0.04, χ^2^ = 4.30, p = 0.038). These results suggest that the different PCC moderation roles in the ASD vs NT groups might be equivalent to its role in the self-perceived autistic traits.

## Discussion

In the present study, we conceptualized similarity–affiliation as a multi-level coupling system and tested whether ASD differentially alters selective context-dependent and global trait-like components of this process. Our findings showed that ASD did not disrupt the fundamental tendency to affiliate with relatively similar others. Instead, ASD was characterized by an amplified global coupling between perceived similarity and affiliative orientation, suggesting a greater reliance on similarity as a stable heuristic for social engagement. Specifically, when perceived similarity is high, affiliative responses were comparable to NT individuals. Group differences only emerged when similarity was low, where affiliation declined more sharply in ASD, indicating a reduced tendency to engage beyond similarity boundaries. On the other hand, NT individuals could still affiliate depending on context even if their overall perceived similarity was low. This pattern was consistently observed across two independent samples that varied in demographic composition, self-perceived autistic traits, and data collection methods, supporting the robustness of the effect. Our findings suggest that autistic individuals may not show reduced social motivation; instead, they may show a more selectively gated and less context-flexible pattern of social interaction.

At the selective level, our findings showed that when an interaction partner is perceived as more similar than one’s typical baseline, affiliative behaviors increase comparably across ASD and NT groups, consistent with meta-analytic evidence^18^. Thus, ASD-related differences may not lie in the direction of similarity-based responding, but rather in downstream processes, such as translating attraction into enacted approach, sustaining interaction under social-cognitive load, or navigating higher-cost social contexts. This interpretation is consistent with the overall lower affiliative behavior observed across our ASD samples compared with their NT counterparts when they reported the same level of perceived similarity. We also replicated prior evidence that perceived control moderated similarity attraction in NT individuals^22^, indicating context-sensitive social tuning. For NT individuals, affiliation may partially function as a risk-management strategy: when perceived control is low, affiliating with similar others may provide emotional and instrumental resources, as similarity is associated with cooperation and trustworthiness^51,52^. However, this effect was absent—and not meaningfully present—in ASD, suggesting reduced context sensitivity rather than a disruption of similarity attraction per se.

In contrast, at the global level, trait empathy selectively shaped similarity–affiliation alignment in ASD but not NT individuals, a pattern replicated across samples. Notably, this effect did not reflect differences in mean levels of affiliation or perceived similarity, but rather in the degree to which two tendencies co-varied at the trait level. Social interaction and mentalizing can be cognitive demanding and unpredictable, especially for individuals with ASD, who often show a preference for predictability and systematic processing^28^. Perceived similarity may therefore serve as a stable, rule-based scaffold for interaction, reducing reliance on inferring others’ fluctuating mental states. Individuals with lower empathic resonance may rely more heavily on similarity as a heuristic cue for affiliation, strengthening the global coupling between similarity perception and overall affiliative orientation. Consistent with prior suggesting altered self-other distinction in autism (e.g., ^7–9^, null findings see ^53,54^), reduced empathic engagement may increase reliance on self-referential processing—using oneself as a model to interpret others—as a compensatory strategy. Importantly, the moderating role of trait empathy was specific to clinically diagnosed ASD rather than to elevated self-reported autistic traits, echoing previous work from our lab that diagnosed autistic individuals exhibit distinct social patterns in naturalistic contexts^6^.

Furthermore, although correlational in nature, our findings suggest distinct roles of empathy in shaping similarity–affiliation coupling across ASD and NT individuals. In NT participants, although the moderation effect of trait empathy did not reach statistical significance, higher trait empathy was descriptively associated with stronger similarity–affiliation coupling at both within- and between-person levels. This pattern aligns with theoretical accounts proposing that empathy enhances social attunement and strengthens the translation of perceived similarity into affiliative behavior^31^. In contrast, this pattern appeared reversed in ASD individuals, the underlying mechanisms of which warrant further investigation.

Biological plausibility of mechanistic dissociation at the global similarity-affiliation alignment across ASD and NT groups was further supported by our neural data. Consistent with accounts positioning the hippocampus as a hub for relational binding and flexible reuse of social information, we found that larger hippocampal volume predicted a stronger relationship between perceived similarity and affiliative behavior in NT individuals. A larger hippocampus may enhance the encoding and recombination of self-other relational features (e.g., shared traits, preferences, histories), allowing perceived similarity to be rapidly integrated with prior experiences and deployed to motivate affiliation. Damage to, or atypical development of, the hippocampus yields inflexible, stereotyped choices precisely when tasks demand generating, updating, and flexibly using social information (e.g., character judgments, conversational tracking, empathy/perspective-taking)^46^. In autism, converging evidence indicates atypical hippocampal structure and development (e.g.,^55,56^, for null finding see^57^), which could weaken relational binding for social information. Therefore, similarity may not be routed through relational/episodic integration, reducing hippocampal involvement in affiliation decisions. Combined with ASD’s documented shift toward perceptual-level processing and reduced altercentric influence^58^, affiliation may rely more on surface or perceptual cues or non-hippocampal-involved heuristics, explaining the absent hippocampal moderation in ASD.

Differential PCC-mediated mechanisms underlying similarity attraction emerged in ASD vs NT groups. In ASD, smaller PCC volume was associated with stronger global similarity-affiliation alignment, consistent with the idea that when the neural machinery that computes graded social distance is attenuated, individuals rely more on surface similarity as a low-cost proxy for closeness. This pattern aligns with accounts of ASD emphasizing reduced integration of social context and atypical network organization within social and mentalizing systems^59,60^. Smaller PCC may bias behavior toward simpler, perceptual similarity heuristics, yielding a stronger similarity–affiliation coupling despite weaker map-based control. By contrast, among NT participants, larger PCC volume predicted stronger similarity–affiliation coupling—suggesting that a more robust PCC supports richer, more precise social-distance mapping and allows similarity to be selectively up-weighted when it legitimately signals safety, trust, or shared norms. Together with prior work showing that PCC and precuneus tracks the vector length of social space while hippocampus encodes relational coordinates^41–43,45^, these results point to PCC as a control signal over social proximity: when intact and well-resourced (larger PCC in NT), it endorses similarity when appropriate; when reduced (smaller PCC in ASD), it leaves behavior more heuristic-driven, making similarity a dominant—and sometimes overgeneralized—driver of affiliation. This double dissociation accords with our hypotheses linking the hippocampus to relational integration and the PCC to social-distance mapping, providing convergent biological evidence for distinct affiliative architectures in autism and neurotypicality.

Our work has several limitations and addressing them may prove fruitful for future research. First, our design is correlational and therefore does not permit causal inference. Although this study offers a theory-driven examination of both selective and global level similarity–affiliation coupling, future experimental research is needed to directly test the underlying mechanisms. For instance, experimentally manipulating empathy (e.g., through perspective-taking induction) or perceived control (e.g., via controllability framing) could clarify their causal roles in moderating similarity–affiliation coupling. Similarly, perceived similarity could be manipulated within individuals (e.g., varying target similarity across trials) to test its immediate impact on affiliative behavior, and between individuals (e.g., assigning participants to high- vs. low-similarity social environments) to examine its broader influence on trait-level affiliative orientation.

Second, the observed effect sizes were small across groups. However, this pattern is consistent with prior work in non-autistic populations (e.g.,^22^) and aligns with evidence that social-cognitive differences between autistic and non-autistic are often subtle yet reliable (e.g.,^9^). Thus, we view these effects as meaningful, as even small effects can accumulate across time, situations, or individuals and may therefore exert substantial influence in the long run^61^. Third, empathy is a multifaceted construct, encompassing cognitive, affective, and social skills components. Our measure combined these dimensions, which limited our ability to isolate their distinct contributions. We also focused exclusively on trait empathy, whereas empathy is highly context-sensitive^62^. Future studies should differentiate between cognitive and affective empathy and examine both trait and state aspects.

Another limitation concerns the construct of “perceived similarity.” We did not examine how similarity varied as a function of different dimensions (e.g., attitudes, appearance, or behavioral styles) and how these dimensions might differentially shape affiliation in ASD versus NT individuals. Also, consistent with prior studies, we examined perceived similarity because in prior studies it more strongly predicted interpersonal attraction than objective similarity^18,23,63–65^, as it’s driven by how we interpret the information available about others. These remain important questions for future research, particularly when integrated with functional neuroimaging to examine brain regions engaged during real-time similarity judgments, rather than relying solely on structural measures. Finally, although our multiple samples strengthen confidence in the findings, future research should extend and replicate this work to more diverse populations—culturally, socioeconomically, and across the full autism spectrum, including individuals with intellectual disability.

Nevertheless, our study makes several notable contributions. Our findings extend the similarity-affiliation coupling phenomenon from discrete, one-off self-report ratings of affiliation to ecologically validated, naturalistic measures of social behavior. Our results underscore the need to distinguish between liking someone (affective attraction) and choosing to affiliate (behavioral attraction), which doesn’t always align^24^, especially in autism. With replicated and validated multilevel analyses showing distinct mechanisms in ASD, our study demonstrates a multi-mechanism model in which different cognitive mechanisms (empathy- vs. control-based) and neural architectures can yield the same affiliative behaviors. Another strength of our samples was their balanced sex representation, including an unusually high proportion of autistic women, thereby addressing a longstanding gap in the literature on female autism^66^. In sum, our results revealed unique social-cognitive mechanisms in ASD and highlight the importance of understanding how ASD individuals affiliate and form bonds with others at both selective and global levels of behaviors.

More broadly, these findings point to a shift in the architecture of social behavior in ASD: from flexible, context-sensitive modulation toward a more rule-based and stable organization centered on similarity. This interpretation aligns with our findings of reduced sensitivity to contextual cues such as perceived control, alongside a stronger reliance on trait-level factors. Conceptually, this reframing may move the field away from deficit-based accounts of social motivation in autism and toward a model in which social engagement is structured differently—more predictable, similarity-guided, and less dependent on fluctuating contextual inputs. Such a perspective has important implications for understanding social functioning in autism, suggesting that interventions may benefit from leveraging perceived common ground and explicitly scaffolding bridges across perceived dissimilarity, rather than assuming a lack of social drive per se.

## Methods

### Participants

#### Online adult sample

We recruited a large adult ASD participant sample through the Simons Powering Autism Research (SPARK) Research Match program, a project supported by the Simons Foundation. All registered SPARK participants self-attested to having received professional diagnoses of ASD. The eligibility criteria were: (1) ages 18 to 30, (2) Social Communication Questionnaire^67^ total score > 9. Among 956 participants who consented, 675 individuals completed all surveys and the social task. The sample size after excluding 100 participants with an “ASD validity flag” (criteria include diagnosis rescinded, or diagnosis age earlier than 15 months, repetitive behavior scale-revised score < 10, unknown diagnosis source, etc.) identified in the SPARK phenotype dataset was 575 (409 females). IQ was measured by cognitive tests from TestMyBrain (https://www.testmybrain.org). The Icahn School of Medicine at Mount Sinai’s Institutional Review Board approved the study protocol. All participants provided written informed consent and received compensation for their participation.

Neurotypical (NT) controls were recruited in the United States from Prolific (www.prolific.com), a platform that helps researchers recruit participants for their online research. The exclusion criteria were: (1) neurodevelopmental diagnoses, including ASD, dyslexia, attention deficit disorder or attention deficit hyperactivity disorder, (2) mild cognitive impairment or dementia, (3) history of neurological concerns such as epilepsy, traumatic brain injury, Parkinson’s disease, epilepsy, seizures, or multiple sclerosis, (4) other chronic diseases such as diabetes, heart disease or stroke, and (4) first language is not English. IQ was measured by ICAR (The international cognitive ability resource https://icar-project.com).

Among the final ASD sample of 575, 200 had self-identified cognitive or language delays (such as intellectual disability, specific language impairment). We matched the NT sample to the ASD sample by removing those who self-reported cognitive or language delays. From a total sample size of 696 NT participants, we selected 357 participants who matched our ASD sample based on sex, age, and education using the “MatchIt” package in R.

#### In-person adult sample

ASD participants were recruited through physical flyers around New York City, email listserv announcements, local research registry and word of mouth. The eligibility criteria were: (1) ages 18 to 50, (2) meeting DSM-5 criteria for ASD, (3) having an IQ over 60 (assessed onsite by the Wechsler Abbreviated Scale of Intelligence and Wechsler Intelligence Scale for Adults^68,69^), (4) having no history of neurological concerns like epilepsy or traumatic brain injury, and (5) no substance or alcohol abuse disorders no recreational drug use. ASD screening was conducted by licensed clinicians using the Autism Diagnostic Observation Schedule, 2nd edition (ADOS-2^70^), supplemented with developmental and clinical history as needed, to inform DSM-5 criteria. We intentionally over-recruited females to ensure better representation of this underrepresented group in autism research.

NT participants were recruited through announcements posted on physical flyers around New York City and email listservs with the eligibility criteria of (1) age between 18 and 50, (2) IQ >60 (assessed via The Wechsler Abbreviated Scale of Intelligence; WASI-II), (3) no 1st degree relatives with idiopathic ASD, (4) no history of neurological concerns (e.g., epilepsy, TBI), and (5) no recreational drug use 24 hours prior to MRI visit.

One hundred and sixteen autistic individuals with onsite clinically confirmed ASD and 125 NT individuals participated in the study. Among these participants, 103 ASD and 122 NT for ASD completed the social navigation task with valid data (if they completed at least 75% of the trials and demonstrated above-chance accuracy on the post-task memory test) as well as provided demographic data. Twenty-two participants from the ASD group and 5 participants from the NT group did not complete MRI scanning, and 7 participants’ data were removed in the ASD group due to an unusable structural scan. To be consistent with our previous study sample (Hao et al., in review), for neuroimaging analysis, two participants with ASD were further removed due to lack of IQ measure. After applying these criteria, the final sample consisted of 72 sex-balanced (52% female) adults with ASD and 102 NT (66% females) for volumetric analysis. Our structural neuroimaging data included all data from the previous study that examined social anxiety in ASD, but the hippocampus and PCC volumes were not analyzed in the previous study^71^. The Icahn School of Medicine at Mount Sinai’s Institutional Review Board approved the study protocol. All participants gave written informed consent and received compensation for their participation.

#### Online adolescent sample

Along with online adult data collection for autistic adults, we also collected an autistic adolescent sample through the SPARK Research Match program. Eligibility criteria for adolescents were: (1) ages greater than 12 and less than 18, (2) Social Communication Questionnaire^67^ total score > 9. Among 972 participants who consented, 445 individuals completed all surveys and the social task (this sample included those with cognitive delays). All parents provided written informed consent and received compensation for their participation.

### Behavioral and cognitive measures

#### Affiliative behaviors

In this study, participants engaged with a role-playing game designed to map out their individual social space, tracing their interactive pathways with various characters^41^. We have two version of the game for data collection in online and in-person samples. For online samples (adults and adolescents), participants started a new school and needed to find their way around and settle in. Their task was to join a club and find a locker at their new school. For the in-person adult sample, participants find themselves in a new town, with the objective of securing employment and housing by interacting with local residents. Both tasks were designed to parallel each other in the number and types of interactions and the roles of the characters. During gameplay, participants encountered different social characters, with conversation occurring via text bubbles (Fig.1A). The characters possessed distinct attributes suggestive of their social roles—such as an old acquaintance, an assistant, or a potential employer. For the participants navigated the social scenarios by choosing from two dialogue options, using a button press to dictate their responses. Some of these responses represent *affiliative behaviors*, with one option representing higher affiliative behaviors (= +1) and the other option representing lower affiliative behaviors (= −1). On the other hand, affiliation trials were intermixed with trials representing *power* behavior: either being authoritative (= +1) or submissive (= −1). Apart from an example affiliation trial in Figure 1A, some example affiliation trials are: You say “So, what’s the deal with Maya? Catch me up! (+1) or you continue looking at your phone (−1). Chris goes in for a hug. You shake his hand instead (−1) or you hug him for a long moment (+1). Some examples of power trials are: You wait for Chris to take the middle seat (+1) or you immediately move over to the middle seat (−1). Maya says to you: “there is a phone number on their website.” You go to the website on your phone (−1) or you say, “give them a call and schedule a meeting, please (+1).” This interactive format allows participants to experience a consistent narrative while their choices actively influence the direction of the story, akin to “choose-your-own-adventure“ games. There are 5 characters in the game, such as boss or assistant. For each character, there are 6 trials of affiliation choices and 6 trials of power choices. Three additional trials involved neutral interaction with a control character. Affiliative behaviors were calculated by averaging affiliation choices over all decision trials and across the five social characters during the social navigation task to derive a person-level measure of affiliative tendency, which was used in the similarity attraction analysis.

The items in our in-person version naturalistic task have been validated and examined across multiple samples^6,41–43,72–74^. Our task was intentionally designed to simulate social cognitive maps (i.e., affiliation and power-based social scenarios) that integrate multiple autism phenotype dimensions—including social motivation, reciprocity, compliance, and communication—within realistic social decision-making contexts. By validating individual items against known autism-relevant constructs and documenting robust ASD vs. control group differences, we demonstrate the construct specificity and ecological validity of the task (Hao et al., in review, supplementary materials). Furthermore, the task proved sensitive enough to detect nuanced subgroup differences within the autistic population, such as the influence of depression on affiliation perception or social anxiety on power-related behaviors^71,73^.

#### Perceived social interaction

After completing the task, participants were asked to rate their feelings of affiliation and power. They were instructed that affiliation represented the friendship or intimacy they felt with each character and that power represented the control and dominance they felt each character possessed over them. Participants also rated their perceived similarity to each of the character on a scale of 1 to 100. Perceived affiliation and similarity measures were collected in all samples. In online adult and adolescent samples only, participants rated their perceived control during the social navigation task by rating how much perceived impact they had to each of the character on a scale of 1 to 100.

#### Trait empathy

For the trait empathy measure, we collected the Empathy Quotient developed by Baron-Cohen and Wheelwright^50^. The empathy scale—which includes three dimensions of affective empathy, cognitive empathy and social skills—has been demonstrated to be a valid and reliable measure of empathy and social skill^50,75,76^.

#### Self-rated autism symptoms

Self-rated autism symptoms were measured by the Broad Autism Phenotype Questionnaire (BAPQ), a self-report questionnaire for adults. This questionnaire has succeeded in meeting both sensitivity and specificity requirements for detecting the broader autism phenotype^77^. Its robust psychometric properties^77,78^ and absence of ceiling effects^79^ in individuals with ASD indicate that it performs effectively in both clinical populations^80^ and the general population.

### Neuroimaging acquisition and processing

Structural MRI data was acquired for all participants on a 7 Tesla whole body scanner (Magnetom, Siemens Healthcare, Erlangen, Germany). A SC72CD gradient coil was used with a single coil transmit and a 32-channel head coil (Nova Medical, Wilmington, MA, USA). A T1-weighted MP2RAGE sequence was performed on each participant, with a 0.7 mm × 0.7 mm × 0.7 mm voxel resolution. Field of view (FOV) was 225 × 183, orientation of scan was coronal, repetition time (TR) was 6000 ms and echo time (TE) was 3.62 ms. A coronal-oblique T2-weighted turbo spin echo (T2-TSE) sequence was also obtained for all participants, with a 0.43 mm × 0.43 mm × 2.0 mm voxel resolution. FOV was 222 × 177, orientation of scan was coronal, TR was 9000 ms and TE was 69 ms.

Results included in this manuscript come from preprocessing performed using *fMRIPrep* 22.0.0 (RRID:SCR_016216)^81,82^, which is based on Nipype 1.8.3 (RRID:SCR_002502)^83,84^. One T1-weighted (T1w) image was found within the input BIDS dataset. The T1-weighted (T1w) image was corrected for intensity non-uniformity (INU) with N4BiasFieldCorrection^85^, distributed with ANTs 2.3.3 (RRID:SCR_004757) ^86^ , and used as T1w-reference throughout the workflow. The T1w-reference was then skull-stripped with a *Nipype* implementation of the antsBrainExtraction.sh workflow (from ANTs), using OASIS30ANTs as the target template. Volume-based spatial normalization to two standard spaces (MNI152NLin2009cAsym, MNI152NLin6Asym) was performed through nonlinear registration with antsRegistration (ANTs 2.3.3),using brain-extracted versions of both the T1w reference and the T1w template. The following templates were selected for spatial normalization: *ICBM 152 Nonlinear Asymmetrical template version 2009c*^87^[RRID:SCR_008796; TemplateFlow ID: MNI152NLin2009cAsym], *FSL’s MNI ICBM 152 non-linear 6th Generation Asymmetric Average Brain Stereotaxic Registration Model*^88^[RRID:SCR_002823; TemplateFlow ID: MNI152NLin6Asym]. FreeSurfer (http://surfer.nmr.mgh.harvard.edu) automated segmentation of the volumes were used to extract hippocampus and PCC volumes.

### Statistical analysis

We conducted multilevel analyses using mixed-effects models to account for both within-person and between-person factors. The within-person level examined whether individuals showed greater affiliation toward characters they perceived as more self-similar (five characters per participant). At the between-person level, we tested whether individuals’ overall perception of similarity predicted their overall tendency to affiliate with others. To separate these two levels, within-person similarity scores were group-mean centered, ensuring independence from the between-person factor. This allowed us to estimate both within- and between-person effects simultaneously (equation see below). All models included covariates for age, sex, character role, and socioeconomic status (SES; composite of income and education). Continuous predictors were z-standardized.

Let $A_{ij}$ be affiliation for participant i with character j, and $S_{ij}$ be perceived similarity.

Decompose similarity

$${\overset{‾}{S}}_{i}=\frac{1}{J}\sum_{j=1}^{J}\;S_{ij},\;S_{ij}^{W}=S_{ij}-{\overset{‾}{S}}_{i}$$

Multilevel model

$$A_{ij}=\beta_{0}+\beta_{W}S_{ij}^{W}+\beta_{B}{\overset{‾}{S}}_{i}+u_{0i}+u_{Wi}S_{ij}^{W}+\varepsilon_{ij}$$

Interpretation:

- $\beta_{W}$ : within-person (selective) similarity-attraction effect
- $\beta_{B}$ : between-person (global) similarity-affiliation alignment

Where:

- $u_{0i}$ : random intercept (person-level baseline affiliation)
- $u_{Wi}$ : random slope for within-person effect
- $\varepsilon_{ij}$ : residual

Let $G_{i}$ be group (e.g., 0=NT,1=ASD ).


$$A_{ij}=\beta_{0}+\beta_{W}S_{ij}^{W}+\beta_{B}{\overset{‾}{S}}_{i}+\beta_{WG}\left(S_{ij}^{W}\cdot G_{i}\right)+\beta_{BG}\left({\overset{‾}{S}}_{i}\cdot G_{i}\right)+u_{0i}+u_{Wi}S_{ij}^{W}+\varepsilon_{ij}$$

- $\beta_{WG}$ : group difference in within-person coupling
- $\beta_{BG}$ : group difference in between-person coupling

For the basic mixed-effects model, affiliative behaviors were predicted from perceived similarity, with random intercepts and slopes. In the online adult sample, including random slopes significantly improved model fit compared to random intercept-only models (p = 0.003), so random slopes were retained. In the in-person adult sample, random slopes did not improve model fit (p = 0.321), so only random intercepts were included. In the online adolescent sample, random slopes did not improve model fit (p = 0.055), so only random intercepts were included.

All statistical tests were two-sided. For each analysis, we reported test statistics, standardized beta coefficients (where applicable), and *p*-values (adjusted by false discovery rate). We also reported semipartial R^2^ and simple slope analysis for effect sizes.

Research Question Models:

- Model 1: We extended basic model by adding an interaction between similarity and diagnostic group (ASD vs. NT) to predict affiliation, including the above covariates in both online and in-person adult samples. We also explored the effect for self-rated autistic symptoms for both online and in-person adult samples.
- Model 2: We extended the basic model with a three-way interaction among perceived control, similarity and diagnostic group. For post-hoc tests, we examined two-way interactions of perceived control and similarity separately within each group. This was done in online adult and online adolescent samples.
- Model 3: We added a three-way interaction among trait empathy, perceived similarity and diagnostic group, again followed by within-group two-way interaction post-hoc analyses. This was done in both online and in-person adult samples. To validate the empathy effect, we conducted sensitivity tests by adding those online autistic adults with cognitive delays. We also conducted specificity tests by conducting same analysis for self-rated autistic symptoms for both online and in-person adult sample. We also tested it in an online adolescent ASD sample.
- Model 4: We replaced empathy with hippocampal volume or PCC volume in the in-person adult sample to test three-way interaction among brain region, perceived similarity and diagnostic group. This was followed by specificity test by examining self-rated autistic symptoms. Bilateral hippocampal and PCC volumes were averaged for statistical analysis. We performed ROI analysis with these two regions; however, we also did exploratory whole-brain analysis with FDR correction to see if other brain regions emerge as significant moderators for similarity attraction effect.

To complement our frequentist null-hypothesis significance testing framework, we also conducted Bayesian statistical inference to evaluate evidence for the absence of a practically meaningful interaction between perceived control and similarity in the ASD sample. Specifically, we fit Bayesian multilevel models corresponding to the frequentist models using the *brms* package in R. Weakly informative priors were specified for fixed effects (normal distributions centered at zero, SD = 0.3). To assess practical equivalence, we defined a smallest effect size of interest (SESOI) of ±0.05 on standardized regression coefficients and quantified the proportion of posterior mass falling within this region of practical equivalence (ROPE). In addition, we computed interval-null Bayes factors comparing the hypothesis that the interaction effect lay within the ROPE (|β| ≤ 0.05) against the alternative that the effect was non-trivial (|β| > 0.05), using an interval Savage–Dickey density ratio. Bayes factors (BF_01_) greater than 10 were interpreted as strong evidence for practical equivalence. For group-specific effects, posterior distributions for the ASD interaction were derived directly from the fitted three-way interaction model.

## Figures and Tables

**Table 1.** Statistics of demographic information and main research variables. Welch two-sample t-tests were performed to evaluate group differences of continuous variables between the ASD and NT samples. Two-Proportion Z-Tests were performed for proportion comparisons. Significant differences are bolded.

A. Online adult	ASD (n = 357)	NT (n = 357)	test
Sex	M 84 F 273	M 93 F 264	χ^2^ = 0.61, p = 0.435
Age	25.33 (3.25) 18.50 – 30.92	25.01 (3.40) 18.00 – 34.00	t = 1.28, p = 0.202
Education	College and above 129 High school and below 228	College and above 187 High school and below 170	t = −1.36, p = 0.173
# Self-rated autism symptom severity	145.52 (22.95) 96 – 211	113.00 (21.85) 50 – 189	**t = 19.10, p < 0.001**
Affiliative behaviors	0.13 (0.30) −0.80 – 0.80	0.33 (0.25) −0.53 – 0.73	**t = −9.86, p < 0.001**
Perceived similarity	39.74 (15.89) 1 – 100	48.62 (13.98) 1.8 – 90	**t = −7.93, p < 0.001**
Trait empathy	10.82 (6.67) 0 – 34	22.36 (8.27) 2 – 40	**t = −20.53, p < 0.001**
Perceived control during social navigation task	55.50 (13.78) 1 – 88	59.20 (12.16) 13.5 – 100	**t = −3.80, p < 0.001**

B. In-person adult	ASD (n = 103)	NT (n = 122)	test
Sex	M 55 F 48	M 42 F 80	**χ^2^ = 8.20, p = 0.004**
Age	26.69 (7.80) 18.00 – 50.36	28.85 (8.36) 18.87 – 54.01	**t = −2.00, p = 0.047**
Education	College and above 61 High school and below 42	College and above 113 High school and below 9	**χ^2^ = 35.54, p < 0.001**
Self-rated autism symptom severity	138.75 (25.31) 56 – 189	99.48 (22.21) 44 – 153	**t = 12.26, p < 0.001**
Affiliative behaviors	0.15 (0.36) −0.85 – 0.85	0.37 (0.24) −0.31 – 0.92	**t = −5.18, p < 0.001**
Perceived similarity	40.65 (18.03) 1.00 – 90.80	42.92 (11.74) 1.00 – 65.40	t = −1.10, p = 0.274
Trait empathy	11.13 (8.13) 0 – 35	25.00 (8.54) 4 – 40	**t = −11.83, p < 0.001**
* Bilateral hippocampal volume (mm^3^)	3114 (511) 1521 – 4221	3134 (515) 823 – 4124	t = −0.26, p = 0.796
* Bilateral PCC volume (mm^3^)	2996 (524) 1311 – 4369	3180 (487) 2211 – 4851	**t = −2.35, p = 0.020**

C. Online adolescent ASD	ASD (n = 445)
Sex	M 355 F 90
Age	14.70 (1.56) 12.17 – 17.92
Affiliative behaviors	0.11 (0.33) −0.80 – 0.80
Perceived similarity	45.54 (16.52) 1 – 92
Trait empathy	12.91 (7.45) 0 – 40
Perceived control during social navigation task	57.57 (14.34) 1 – 100

Note:

# Self-rated autism symptom severity was measured by Broad Autism Phenotype Questionnaire (BAPQ, 68% of the ASD sample completed it).

Note:

* We computed a bilateral hippocampal (PCC) measure by averaging left and right hippocampal (PCC) volumes. Due to missing values, *the sample sizes for hippocampus and ACC volumes are 72 for ASD and 102 for NT*

## Data Availability

Data and code for this study is available at osf.io/f4uhc
